# King Edward's Hospital Fund

**Published:** 1902-11-29

**Authors:** 


### THE INSTITUTIONAL WORKSHOP.

### KING EDWARD'S HOSPITAL FUND.

### THE AMALGAMATION OF SMALL HOSPITALS.

#### Grants Awarded.

A Meeting of the General Council of King Edward's Hospital Fund for London for the purpose of awarding grants to the hospitals for the present year was held on the 24th inst. at York House, St. James's Palace. The Prince of Wales was in the chair. Those present were Viscount Duncannon, Lord Rothschild, Lord Rowton, Lord Lister, Lord Reay, the Lord Mayor of London (Sir Marcus Samuel), the Bishop of London, Sir Trevor Lawrence, Sir William Church, Sir H. G. Howse, Sir John M'Dougall, Sir William Collins, Sir Augustus Prevost, Sir Joseph Dimsdale, Sir Savile Crossley, Sir Henry Burdett, Mr. Albert G. Sandeman, Mr. Frederick M. Fry, Mr. C. Stuart-Wortley, Mr. T. Burt, M.P. Mr. J. G. Craggs, the Rev. J. Guinness Rogers, the Chief Rabbi (Dr. Adler), Mr. Hugh C. Smith, and Mr. Latham, K.C.

The minutes of the last meeting were read, confirmed, and signed, and letters of regret were read from the following: the Duke of Bedford, the Duke of Norfolk, the Bishop of Rochester, Cardinal Vaughan, the Rev. Dr. Bowman Stephenson, Lord Iveagh, Lord Farquhar, the Postmaster-General, Sir John Aird, Mr. Sydney Buxton, Mr. Hambro, Mr. J. Wernher, and the Prime Warden of the Fishmongers' Company.

Mr. H. C. Smith presented the report of the Executive Committee.

#### Report of the Distribution Committee.

Lord Lister read the report of the Hospitals' Distribution Committee, as follows: The committee humbly desire to congratulate his Royal Highness, the president of the Fund, on the success that has attended it during the first year of his tenure of office. In spite of the unusual demands on the charity of the public entailed by the war and its consequences, the pecuniary support received in the shape of subscriptions and donations during the last 12 months has greatly exceeded that of any previous year. The sum available on the present occasion has been further increased by the fact that his Majesty the King has shown his continued great interest in the fund which he founded by being graciously pleased to transfer to it the Coronation gift of his subjects. The Executive Committee were thus able, with the approval of his Royal Highness the President, to recommend the distribution for this year of £100,000, which is double the amount of last year's grants. In considering how the additional sum may be bestowed to the best advantage with a view to the permanent good of the hospital, the committee decided to recommend that it should be devoted to four principal objects, viz.:

1. The opening of closed beds.
2. The wiping out or diminution of debt.
3. The carrying out of improvements by building or otherwise; and
4. The supply of various special needs and increased aids to general maintenance.

As regards the first of these objects the committee recommend the opening of 112 closed beds, which will bring up the total of beds opened and maintained by the fund to 433. To opening and maintaining these additional beds we advise that £7,450 should be devoted, which will involve, at least for some time to come, an annual similar payment. Not many beds now remain closed in such of the Metropolitan hospitals as have applied for grants from the fund. Of these some are used for nurses' accommodation or are kept unemployed to facilitate the cleaning of the wards, and of the remainder the committee do not see their way to advise the opening in consequence of unsatisfactory circumstances. The committee hope before another year to make it a condition of continuing the grants for the maintenance of beds opened by aid of the Fund that those beds be really free in the sense of the patients being admitted without reference to subscribers' letters, but simply in accordance with the necessities of the cases.

On the second object, the reduction of debt, we recommend that £29,250 be expended. Grants for this purpose will, in many instances, be a great boon, which will, we trust, result in permanent benefit. The third class includes the carrying out, by building or otherwise, of various improvements urgently needed, in order to bring the institutions concerned into a state of thorough efficiency in accordance with present-day requirements. Of the remaining class of grants, devoted mainly to general purposes, it need only be said that it is proposed to deal with the hospitals, as a rule, in a more liberal manner than ever before. The committee hope to be able to bring about the amalgamation of some small hospitals. And if this attempt should prove successful they would be prepared to recommend the appropriation of a considerable sum to this object, which is important alike in the interests of the institutions concerned and in those of the sick poor. It is suggested that the Distribution Committee be appointed to undertake the needful negotiations. In conclusion, the committee desire to bear testimony to the increasing usefulness of the Fund, not only by supplying in larger and larger measure the pecuniary needs of the hospitals, but also by stimulating them to progressive improvement. This is in great measure the result of the self-denying labours of the Visiting Committee, to whom very cordial thanks are due. It is satisfactory to know that, while the Fund is flourishing financially, other institutions with similar objects are also prospering. This gives a sure indication that, thanks largely to the influence of the Fund under its royal patronage, the importance of adequately supporting those beneficent establishments, the Metropolitan hospitals, is being increasingly recognised by the public.

#### Prince of Wales's Speech.

His Royal Highness, in moving the adoption of the report, said: My Lords and Gentlemen,—In moving the adoption of the report, I desire to express entire agreement with the recommendations of my committees and approval of the special objects to which, in many instances, the grants are devoted and the conditions attached to them. In acknowledging the congratulations so kindly offered me on the results of my first year's presidency, I rejoice that we have now arrived within measurable distance of the object of its founder, the King. We are deeply grateful to those who have helped us in our successful efforts. The Executive Committee have already referred to the various sources of that success. But I would particularly recall the great achievement of the Coronation Gift Committee, and the persevering efforts of the League of Mercy. As regards individual benefactors, I am sure we all remember the munificent gift of the late Mr. Samuel Lewis, a gift which, by the generous devotion of Mrs. Lewis, has already been placed at our immediate disposal. I have no doubt that this most considerate action has been of the greatest help to us; not only for its own intrinsic value, but for the impetus given by it to the current of noble liberality, not only in this country but throughout the Empire—indeed, throughout the world—for we have even profited by the beneficence of representatives of foreign nations. Mrs. Lewis's gift was followed by the splendid endowments from Lord Strathcona and Lord Mount-Stephen, who have thus extended to King Edward's Fund that open-handed generosity by which they have in Canada created and endowed so many great works of charity. From India we have received the liberal benefactions of the Maharaja of Sindhia and the Maharaja of Jaipur. Turning still nearer home we have received contributions like that of Mr. Speyer and others, and also substantial help from those who have assisted us by gifts in kind.

THE KING'S THANKS.

I am especially glad that the trustees of the London Parochial Charities have again entrusted to us the administration of the £1,000 a year which for some time past they have allotted to convalescent homes. I feel sure that the policy recommended by my committee of devoting these grants chiefly to convalescent homes connected with London hospitals will commend itself not only to the large body of our contributors, but to the citizens to whose benevolence the existence of the London Parochial Charities Trust is due. For in this way the sick poor of the Metropolis are enabled to complete the cure commenced in the wards of its hospitals. I hope you will agree that we have done very well. Personally I am much pleased with the results of the first year in which I have had the honour to occupy the position to which I was nominated by the King, when his Majesty with great regret was compelled to lay down the work connected with the Fund, but over which he still watches with keen interest. But we want another £10,000 a year even to ensure the same amount for distribution next year. May we hope for still more than this. I take this opportunity of saying with what satisfaction I find that our efforts have not in any way damaged the work of the Sunday and Saturday Funds, with which we are in perfect harmony. As the Fund increases in its distributions I trust it will proportionately grow in influence. I look forward to the day when no hospitals will incur any grave expenditure without desiring to lay its proposal before the Fund representatives. I am confident that the position we have established is largely based on the principle originally laid down—that every hospital receiving a grant should be specially visited and reported on by both medical and lay representatives. I am desired by the King to express his best thanks to all who have in a greater or less degree given their valuable time to the carrying out of the many details of his scheme. I am well aware that the economical working of the Fund is due in no small degree to the employment of honorary secretaries and others, whose work one may, indeed, truly say is a labour of love. (Cheers). I beg to move the adoption of the report.

Sir MARCUS SAMUEL, in seconding the adoption of the report, said: I have the privilege to second the adoption of the report so ably moved by the chairman, and I should like to say that every aid will be gladly rendered by the City in favour of any movement that can be introduced for the benefit of this noble object.

THE BISHOP OF LONDON.

The Bishop of LONDON, in supporting the motion, said: I have been asked to support this motion. I do so not only as the Bishop of London, but also as one who has had the privilege of working for nine years in the slums and who has also had experience of seeing the sick poor in their homes and then seeing the difference when carried to the hospitals, and that makes me support the motion with gratitude. It is only those who have been in and out the poorer districts of London day after day who fully realise what good we have done here to-day. I will take for instance two grants made this morning, £1,500 a year to the Consumption Hospital, Victoria Park, and £750 donation. That hospital was shutting up its wards when I was living down there simply for sheer want of money. It is a great thing to think of the wards that have been re-opened in this hospital by the Fund, and there is also the sum of £5,000 a year to the London Hospital, which I am sure is well deserved. I note with pleasure and confirm what Sir Henry Burdett has said in praise of the Poplar Hospital. I express again the thanks of the many workers for the careful judgment the Executive Committee have displayed in putting the report before us and for their recommendations, which are so useful with regard to the various hospitals, and for not neglecting the convalescent homes, which do such good work. We all feel that the interest his Majesty the King and his Royal Highness the Prince of Wales take in the Fund gives a quiver of inspiration to all the workers associated with this Fund.

It was decided that the awards should be paid on December 22nd next.

His ROYAL HIGHNESS then said: I wish, as president, to convey my best thanks to the General Council, the Executive Committee, the Organising Committee, the Right Hon. Sir Joseph Dimsdale, the Visiting Committee, the Hospitals Distribution Committee, the Convalescent Homes Committee, and the honorary officials.

VOTE OF THANKS TO THE PRESIDENT.

Lord ROTHSCHILD, in proposing a vote of thanks to His Royal Highness for presiding, said: It is now my pleasing duty to call on you to carry a vote of thanks to his Royal Highness for so ably presiding over us on this occasion. Grateful as we are to him for welcoming us here to-day, the Hospital Fund and those who benefit by it must be more than grateful to him, because the increased good we are able to do this year is solely due to his Highness's influence in obtaining from those generous contributors the large sums which he has mentioned. I am sure, and I know you will all agree with me, that the fact that his Royal Highness has followed in the footsteps of his illustrious father, who takes a great and deep interest in the sick and poor of the Metropolis, will induce all to follow that example, and to work to their utmost to make this great institution attain a splendid success in the future. (Hear, hear.)

The Rev. J. GUINNESS ROGERS, in seconding the resolution, remarked that there was none of the dead dumbness of statistics in the report of the Fund, but it was instinct with life both as to the origin and the mode as to which the money was to be applied. He did not think it was possible to secure such a result but for the influence which had been exerted by his Majesty the King and which was now followed by the hereditary and honourable succession of the Prince of Wales. He thought it was a pleasure and a pride to every Englishman to think that the Royal Family of this country was identified with such noble schemes.

His ROYAL HIGHNESS, in reply, said: I beg to thank you, my Lords and gentlemen, for the vote of thanks accorded me. It has been a very great pleasure to me to receive you here in my house this morning, and I assure you that the success which I think has attended my first year's Presidency will encourage me to redouble my efforts in the future. (Hear, hear.)

The proceedings then terminated.

### THE KING'S HOSPITAL FUND, 1902.

### DETAILS OF THE AWARDS.

The details of the awards to the various hospitals are given below:—

Alexandra Hospital for Children, Queen Square, Bloomsbury.—£500 donation.

Belgrave Hospital.—£1,000 donation: £500 to building, £500 to discharge existing debts. Committee view removal with great satisfaction.

Bolingbroke Hospital, Wandsworth Common.—£1,500 donation: £500 to discharge existing debts, £500 for general purposes, and £500 for building.

British Lying-in Hospital, Endell Street, St. Giles.—£300 donation: £200 to Nurses' Home, and call attention to high cost of same.

Cancer Hospital, Fulham Road, Brompton.—£200 donation. An excellent institution.

Central London Ophthalmic Hospital, 238a, Gray's Inn Road.—£200 donation, towards rebuilding.

Central London Throat and Ear Hospital, Gray's Inn Road.—£500 donation towards building fund.

Charing Cross Hospital.—£2,500 towards rebuilding fund.

Chelsea Hospital for Women.—£500 donation, £250 to discharge existing debts.

Cheyne Hospital.—£100 in consideration of the fact that curable cases are admitted.

City of London Hospital for Diseases of the Chest, Victoria Park.—£1,500 annual to support 25 beds reopened by this Fund. £750 donation towards Nurses' Home building fund.

City Orthopædic Hospital, Hatton Garden.—£250 annual to maintain 20 beds reopened by this Fund. £500 special donation to pay off loan.

City of London Lying-in Hospital, City Road—£100 donation.

Clapham Maternity Hospital.—£100 special donation towards furnishing new house.

"Dreadnought" Hospital.—£500 annual for general purposes, £1,000 donation for tropical diseases department, £1,000 special donation.

East End Mothers' Home, Commercial Road.—£200 special donation to repay bank.

East London Hospital for Children, Glamis Road, Shadwell.—£1,750 donation for building.

Establishment for Gentlewomen, Harley Street.—£500 donation.

Evelina Hospital.—An excellent institution.

French Hospital, Shaftesbury Avenue.—£100 donation.

General Lying-in Hospital, York Road, Lambeth.—£400 special donation towards expenses of sanitary improvements.

German Hospital, Dalston.—£150 donation.

Gordon Fistula Hospital, Vauxhall Bridge Road.—£100 donation.

Great Northern Central Hospital.—£750 annual to maintain 15 beds reopened by this Fund. £1,250 donation to discharge existing debts.

Grosvenor Hospital for Women and Children, Vincent Square, Westminster.—£200 donation.

Guy's Hospital.—£5,000 annual to maintain 114 beds reopened by this Fund. £1,000 donation to discharge existing debts.

Hampstead Hospital.—£1,250 donation, of which £1,000 is for building fund.

Hospital for Consumption, Brompton.—£1,000 donation to country branch building.

Hospital for Diseases of the Throat, Golden Square.—£200 donation to discharge existing debt.

Hospital for Epilepsy and Paralysis, Portland Terrace, Regent's Park.—£500 donation to building.

Hospital for Sick Children, Great Ormond Street.—£500 annual to maintain sixteen beds reopened by this Fund. £1,000 donation. Again draw attention of authorities to pressing necessity for new out-patients' department.

Hospital for Women, Soho Square.—£250 annual to open six closed beds. £850 annual to open closed wards. Draw attention to reports of visitors, high cost of in- and out-patients, and to cubic space and superficial area necessary for beds. £400 donation to discharge existing debts.

Hospital of St. Francis, New Kent Road.—No grant.

Hospital of St. John and St. Elizabeth, St. John's Wood.—£500 annual, £500 donation to open closed beds. Draw attention to desirability of keeping phthisis cases separate.

Italian Hospital, Queen's Square, Bloomsbury.—£500 annual to open closed beds, £100 donation.

King's College Hospital.—£1,000 annual to meet deficiency, £2,000 donation.

London Fever Hospital.—£1,500 donation, of which £1,000 towards building convalescent home.

London Homœopathic Hospital, Great Ormond Street.—£200 donation.

London Hospital.—£5,000 annual to meet expenditure out of capital made necessary by improvements in the wards. £3,500 donations to discharge existing debts.

London Lock Hospital (Female), Harrow Road.—£1,000 donation, of which £500 to discharge existing debts.

London Temperance Hospital.—£1,000 donation to be used for rebuilding the out-patients' department.

London Throat Hospital, Great Portland Street.—£50 donation towards painting.

Medical Mission Hospital, Canning Town.—£850 donation, of which £750 to building.

Memorial Cottage Hospital, Mildmay Park.—£50 donation.

Metropolitan Hospital.—£500 annual to maintain 10 beds reopened by this Fund; £2,500 donation to discharge existing debts.

Middlesex Hospital.—£1,000 annual to meet deficiency; £1,500 donation to discharge existing debts.

Mildmay Mission Hospital, Austin Street, Bethnal Green.—£250 donation.

Miller Hospital, Greenwich Road.—£1,000 donation, of which £500 is to discharge existing debts.

Mount Vernon Hospital for Consumption.—£1,000 annual to support 15 beds reopened by this Fund; £500 donation.

National Dental Hospital.—No grant.

National Hospital for the Paralysed and Epileptic.—£750 annual to meet deficiency; £1,000 donation towards improvements.

National Orthopædic Hospital, Great Portland Street.—£500 donation to discharge existing debts.

New Hospital for Women, Euston Road.—£500 donation to discharge existing debts.

North-Eastern Hospital for Children, Hackney Road, Shoreditch.—£500 annual towards maintenance; £1,000 donation for new building.

North-West London Hospital, Kentish Town Road.—£1,000 donation; draw attention to the expediency of obtaining a new hospital.

Paddington Green Children's Hospital.—£700 donation, of which £500 is to discharge existing debts.

Poplar Hospital.—The committee view with satisfaction the excellent management, and trust that the closed ward may be opened by the hospital authorities.

Passmore Edwards' Hospital, East Ham.—£50 donation.

Passmore Edwards' Hospital for Willesden.—£100 donation, of which £50 is towards mortuary and £50 to discharge existing debts.

Queen Charlotte's Lying-in Hospital, Marylebone Road.—£1,000 donation to discharge existing debts.

Queen's Jubilee Hospital, Richmond Road, Earl's Court.—No grant.

Royal Dental Hospital of London.—£300 donation to discharge existing debts.

Royal Eye Hospital, St. George's Circus, Southwark.—£100 donation.

Royal Free Hospital.—£750 annual to meet deficiency; £2,000 towards improvements.

Royal Hospital for Diseases of the Chest, City Road.—£800 donation, of which £500 is to building nurses' accommodation.

Royal Hospital for Children and Women, Waterloo Bridge Road.—£1,500 donation, of which £1,000 is to building.

Royal London Ophthalmic Hospital, City Road.—£2,000 annual and £2,500 donation conditional on thirty more beds being opened.

Royal Orthopædic Hospital, 297 Oxford Street.—£250 donation to discharge existing debts.

Royal Westminster Ophthalmic Hospital.—£500 donation to discharge existing debts.

Samaritan Free Hospital, Marylebone Road.—£500 annual towards maintenance, and £250 donation.

St. George's Hospital.—£2,500 donation, of which £1,500 is towards improvement and equipment.

St. John's Hospital for Diseases of the Skin, Leicester Square.—No grant.

St. Mark's Hospital, City Road.—£200 donation, of which £100 is for electric light.

St. Mary's Children's Hospital, Plaistow.—£500 donation.

St. Mary's Hospital, Paddington.—£1,000 annual to meet deficiency; £1,000 donation to discharge existing debts

St. Monica's Hospital, Brondesbury Park.—£100 donation.

St. Peter's Hospital for Stone, Covent Garden.—£100 donation to discharge existing debts.

St. Saviour's Hospital, Osnaburgh Street.—£100 donation.

St. Thomas's Hostital.—£1,800 donation to maintain the beds reopened.

Tottenham Hospital.—£1,000 annual grant to maintain 15 beds reopened by this Fund; £2,000 donation to building.

University College Hospital.—£1,000 annual to support 25 beds reopened by this Fund; £2,500 donation to discharge existing debts.

Victoria Hospital for Sick Children, Queen's Road, Chelsea.—£1,000 donation to building fund.

West-End Hospital for Diseases of the Nervous System.—£250 donation.

Western Ophthalmic Hospital.—No grant. To be reconsidered when proper progress has been made towards collecting the rebuilding fund.

West Ham Hospital.—£300 annual towards maintenance. £2,000 donation, of which £1,400 towards building.

West London Hospital.—£1,500 annual to maintain new beds opened. £1,000 donation to discharge existing debts. £1,000 donation for bathrooms and other sanitary improvements on the east side, conditional on the work being done.

Westminster Hospital.—£750 annual towards deficiency. £2,000 donation to discharge existing debts.

### Convalescent Homes Committee.

Mr. William Latham, K.C., read the report of the Convalescent Homes Committee as follows:

Owing to the continued liberality of the grant made by the trustees of the London Parochial Charities the committee have again £1,000 to distribute. The committee have followed the course adopted in previous years of making out of the limited sum at their disposal fairly substantial grants to a comparatively small number of institutions, and to a great extent they have repeated grants to those before approved of, in preference to varying the distribution.

### The Awards.

The following awards were recommended:

Alexandra Hospital, Convalescent Home at Painswick.—£50.

Charing Cross Hospital, Convalescent Home at Limpsfield, Surrey.—£100.

Chelsea Hospital for Women, Convalescent Home at St. Leonards-on-Sea.—£50.

East London Hospital for Children, Convalescent Home at Bognor.—£100.

King's College Hospital, Convalescent Home at Hemel Hempstead.—£100.

Mary Wardell Convalescent Home, Stanmore, Middlesex.—£100.

Metropolitan Convalescent Institution, 32 Sackville Street, Piccadilly.—£100.

Middlesex Hospital, Convalescent Home at Clacton-on-Sea.—£100.

Mrs. Kitto's Convalescent Home, South Park, Reigate. £50.

National Hospital for the Paralysed and Epileptic, Convalescent Home at East Finchley.—£50.

Paddington Green Children's Hospital, Convalescent Home at Harrow.—£50.

Queen Charlotte's Lying-in-Hospital, Convalescent Home, at 20 Victoria Road, Kilburn.—£50.

St. George's Hospital and Atkinson Morley's Convalescent Hospital, at Wimbledon.—£50.

Victoria Hospital, Convalescent Home, at Broadstairs. —£50.

